# Diverse Roles of Mitochondria in Immune Responses: Novel Insights Into Immuno-Metabolism

**DOI:** 10.3389/fimmu.2018.01605

**Published:** 2018-07-12

**Authors:** Anusha Angajala, Sangbin Lim, Joshua B. Phillips, Jin-Hwan Kim, Clayton Yates, Zongbing You, Ming Tan

**Affiliations:** ^1^Center for Cell Death and Metabolism, Mitchell Cancer Institute, University of South Alabama, Mobile, AL, United States; ^2^Department of Biology, Center for Cancer Research, Tuskegee University, Tuskegee, AL, United States; ^3^Department of Structural and Cellular Biology, Tulane University School of Medicine, New Orleans, LA, United States

**Keywords:** oxidative phosphorylation, fatty acid oxidation, TCA cycle, regulatory T cell, memory T cell

## Abstract

Lack of immune system cells or impairment in differentiation of immune cells is the basis for many chronic diseases. Metabolic changes could be the root cause for this immune cell impairment. These changes could be a result of altered transcription, cytokine production from surrounding cells, and changes in metabolic pathways. Immunity and mitochondria are interlinked with each other. An important feature of mitochondria is it can regulate activation, differentiation, and survival of immune cells. In addition, it can also release signals such as mitochondrial DNA (mtDNA) and mitochondrial ROS (mtROS) to regulate transcription of immune cells. From current literature, we found that mitochondria can regulate immunity in different ways. *First*, alterations in metabolic pathways (TCA cycle, oxidative phosphorylation, and FAO) and mitochondria induced transcriptional changes can lead to entirely different outcomes in immune cells. For example, M1 macrophages exhibit a broken TCA cycle and have a pro-inflammatory role. By contrast, M2 macrophages undergo β-oxidation to produce anti-inflammatory responses. In addition, amino acid metabolism, especially arginine, glutamine, serine, glycine, and tryptophan, is critical for T cell differentiation and macrophage polarization. *Second*, mitochondria can activate the inflammatory response. For instance, mitochondrial antiviral signaling and NLRP3 can be activated by mitochondria. *Third*, mitochondrial mass and mobility can be influenced by fission and fusion. Fission and fusion can influence immune functions. *Finally*, mitochondria are placed near the endoplasmic reticulum (ER) in immune cells. Therefore, mitochondria and ER junction signaling can also influence immune cell metabolism. Mitochondrial machinery such as metabolic pathways, amino acid metabolism, antioxidant systems, mitochondrial dynamics, mtDNA, mitophagy, and mtROS are crucial for immune functions. Here, we have demonstrated how mitochondria coordinate to alter immune responses and how changes in mitochondrial machinery contribute to alterations in immune responses. A better understanding of the molecular components of mitochondria is necessary. This can help in the development of safe and effective immune therapy or prevention of chronic diseases. In this review, we have presented an updated prospective of the mitochondrial machinery that drives various immune responses.

## Introduction

Mitochondria have many fundamental functions such as energy production, providing metabolites for building macromolecules, and aiding in differentiation, apoptosis, and cell cycle. After reviewing recent literature, two mitochondrial functions appeared to be intriguing. *First*, mitochondria and the endoplasmic reticulum (ER) communicate with each other through signaling molecules (1). *Second*, mitochondria are associated with NLRP3 inflammasome activation (2). Mitochondria are localized near the ER to supply energy for protein and lipid synthesis. For example, Bantug et al. have highlighted that ER and mitochondria junction signaling is critical in CD8^+^ memory T cells. Mechanistically, this happens in crosstalk between ER and mitochondria by mammalian target of rapamycin complex 2, AKT (protein kinase B), and glycogen synthase kinase 3β mediated signaling which promotes respiration (Figure 1.9) (3).

**Figure 1:**
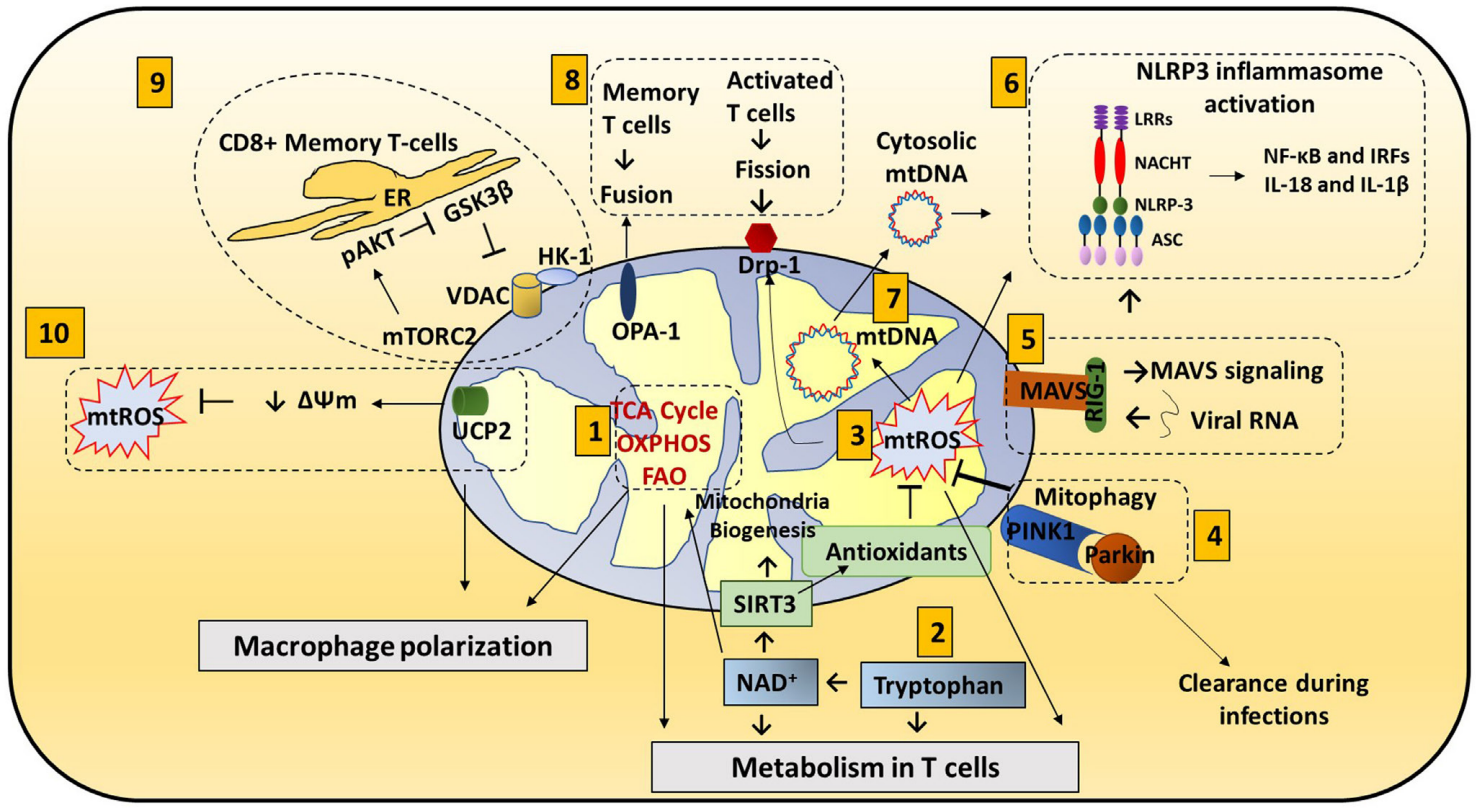
Mitochondria roles in immune responses: mitochondrial components are involved in immune functions. (1) *Metabolic pathways* such as TCA cycle, oxidative phosphorylation (OXPHOS), and fatty acid oxidation (FAO) are important for macrophage polarization and T cell differentiation. (2) *Amino acid metabolism* (Tryptophan metabolism is shown here.) also contributes to mitochondrial immune functions. Tryptophan depletion can cause immune tolerance. NAD^+^ is synthesized from tryptophan. NAD^+^ contributes to SIRT3-mediated mitochondrial biogenesis. In addition, NAD^+^ is required for metabolic pathways and CD4^+^ T cell differentiation. (3) *Mitochondrial ROS* (*mtROS*) control immune cell transcription, metabolism, and NLRP3 mediated inflammation. Antioxidants such as glutathione balance mtROS. SIRT3 is involved in regulating many mitochondrial proteins such as IDH2 (TCA cycle), MnSOD [reactive oxygen species (ROS) balance], and FOXO3. SIRT3 inhibits mtROS. (4) *Mitophagy* is crucial for removing damaged mitochondria. Damaged mitochondria are a source of mtROS so mitophagy balances mtROS. Pink1, localized on the mitochondrial outer membrane, binds to parkin and initiates mitophagy. Parkin mutations can increase the susceptibility to the intracellular bacteria *Mycobacterium leprae* and *Salmonella enterica*. Decreased mitophagy results in increased ROS which further increases the susceptibility to infections. Hepatitis B and C viruses use mitophagy to their benefit. These viruses protect themselves from mitochondria induced apoptosis by activating mitophagy. (5) Mitochondrial antiviral signaling (*MAVS*) in the mitochondria membrane initiates the inflammatory response. Upon viral infections, viral RNA is sensed by RIG-I. RIG-I activates MAVS (located on the outer membrane of mitochondria). MAVS signaling can activate NLRP3 inflammasome and NF-κB/IRF transcription. (6) *NLRP3 inflammasome* contributes to the activation of caspase-1 and leads to NF-κB signaling and production of IL-1β and IL-18. (7) *Mitochondrial DNA* (*mtDNA*) can be released from mitochondria into the cytosol and activate the NLRP3 inflammasome and production of IL-1β and IL-18. Also, mtROS can induce mtDNA mutation. (8) *Mitochondrial dynamics*, fission (OPA-1) and fusion (Drp1), is associated with activated T cells and memory T cells, respectively. (9) *ER and mitochondria junction*: in CD8^+^ memory T cells, mitochondria are placed near the ER. HK-1 (hexokinase-1, an important enzyme in glycolysis) in conjunction with pAKT, mammalian target of rapamycin complex 2 (mTORC2), and glycogen synthase kinase 3β (GSK-3β) mediated signaling cascades regulate CD8^+^ memory T cell metabolism. (10) *Uncoupling protein 2* (*UCP2*), a mitochondrial membrane protein, allows protons to enter the mitochondrial matrix, thereby decreasing mitochondrial membrane potential which further decreases mtROS.

*Immune response and metabolism are closely dependent on each other*. During immune response, immune cells transition from metabolic quiescence to active phase. This transition is associated with a metabolic shift from catabolic to anabolic state. During the quiescence state, macromolecules undergo catabolic pathways to produce energy and support long-term survival. During the anabolic state, macromolecules are synthesized and support a balance between the need for ATP and required metabolites. Depending on the need, immune cells choose a specific pathway such as β-oxidation to generate more ATP than glycolysis. Thus, ATP and metabolic intermediates provide the signals to activate immune responses (4–6).

Hence, mitochondria, the chief organelle for metabolism, have emerged to play crucial roles in the maintenance and establishment of immune responses. Mitochondrial machinery such as metabolic pathways, amino acid metabolism, antioxidant systems, mitochondrial dynamics, mitochondrial DNA (mtDNA), mitophagy, and mitochondrial ROS (mtROS) are crucial for immune functions. In this review, we have presented an updated prospective of the mitochondrial machinery that drives various immune responses.

## Metabolic Pathways are Tightly Controlled in Immune Cells

Mitochondrial metabolic events can have tremendous impact on immune cell function. These metabolic events could be guided by the internal signals from the cell itself or influenced by other surrounding cells. Here, we have pointed out how oxidative phosphorylation (OXPHOS), fatty acid metabolism, and amino acid metabolism in mitochondria influence immune cell activity (Figure 1.1).

### OXPHOS Affects Immune Cell Activity

#### M1 and M2 Polarization

**Figure 2:**
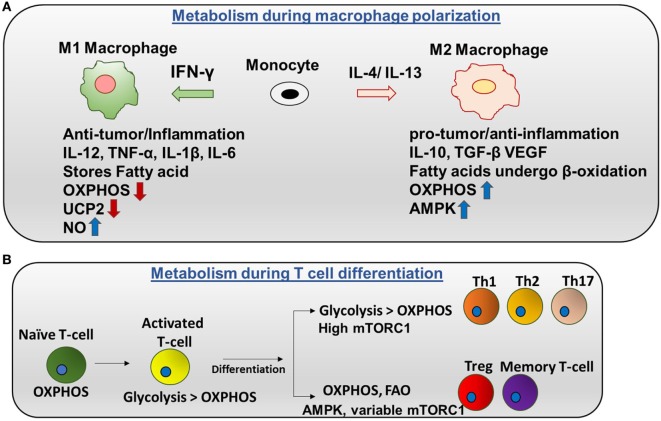
Metabolic regulation in macrophages and T cells: **(A)** metabolism during macrophage polarization: polarization of monocytes to M1 or M2 phenotypes is controlled by the cytokines produced by other immune cells. *M1 macrophages* are activated by IFN-γ produced by Th1 cells. M1 macrophages tend to store surplus FA as triacyclglycerols and cholesteryl esters in lipid droplets, and they exhibit higher aerobic glycolysis and lower oxidative phosphorylation (OXPHOS). Nitric oxide production is higher in M1. Uncoupling protein 2 (UCP2) expression is decreased in M1 macrophages. Contrarily, *M2 macrophages* are activated by IL-4 or IL-13 to regulate anti-inflammation and promote Th2 response and tissue repair. M2 macrophages adopt a metabolic program dominated by fatty acid-fueled OXPHOS and channel FA toward re-esterification and β-oxidation. Silencing UCP2 impairs M2 macrophage activation by IL-4. High adenosine monophosphate-activated protein kinase (AMPK) and low NO is the reason for high OXPHOS in M2 macrophages. **(B)** Metabolism during T cells differentiation: naïve T cells are dependent on OXPHOS as their primary metabolic pathway. By contrast, activated T cells exhibit higher glycolysis than OXPHOS. After differentiation, Th1, Th2, and Th17 have higher glycolysis than OXPHOS and high mTORC1 activity. Memory T cells and regulatory T cells undergo AMPK-dependent FAO and have variable mTORC1.

There are two categories of macrophages—M1 (classically activated) and M2 (alternatively activated). Polarization of monocytes to M1 or M2 phenotypes is controlled by the cytokines produced by other immune cells. M1 macrophages are activated by IFN-γ produced by Th1 cells and lipopolysaccharide (LPS). Plus, M1 exhibits nitric oxide (NO) production (7) and a pro-inflammatory phenotype. Contrarily, M2 macrophages are activated by IL-4 or IL-13 to regulate anti-inflammation and promote Th2 response and tissue repair (Figure 2A) (8).

#### Uncoupling Protein 2 (UCP2) and Macrophage Polarization

Mitochondrial UCP2 is localized in the mitochondrial inner membrane and shuttle protons toward the matrix (Figure 1.10). There is increasing evidence supporting that UCP2 controls mitochondria derived reactive oxygen species (ROS). UCP2 can also influence polarization of macrophages. UCP2 expression is decreased in M1 macrophages. By blocking UCP2, there is a decrease in IL-4 induced M2 macrophage activation (9). However, how UCP2 is regulated in other immune cells is not well elucidated.

#### TCA Cycle in M1 Macrophages

**Figure 3:**
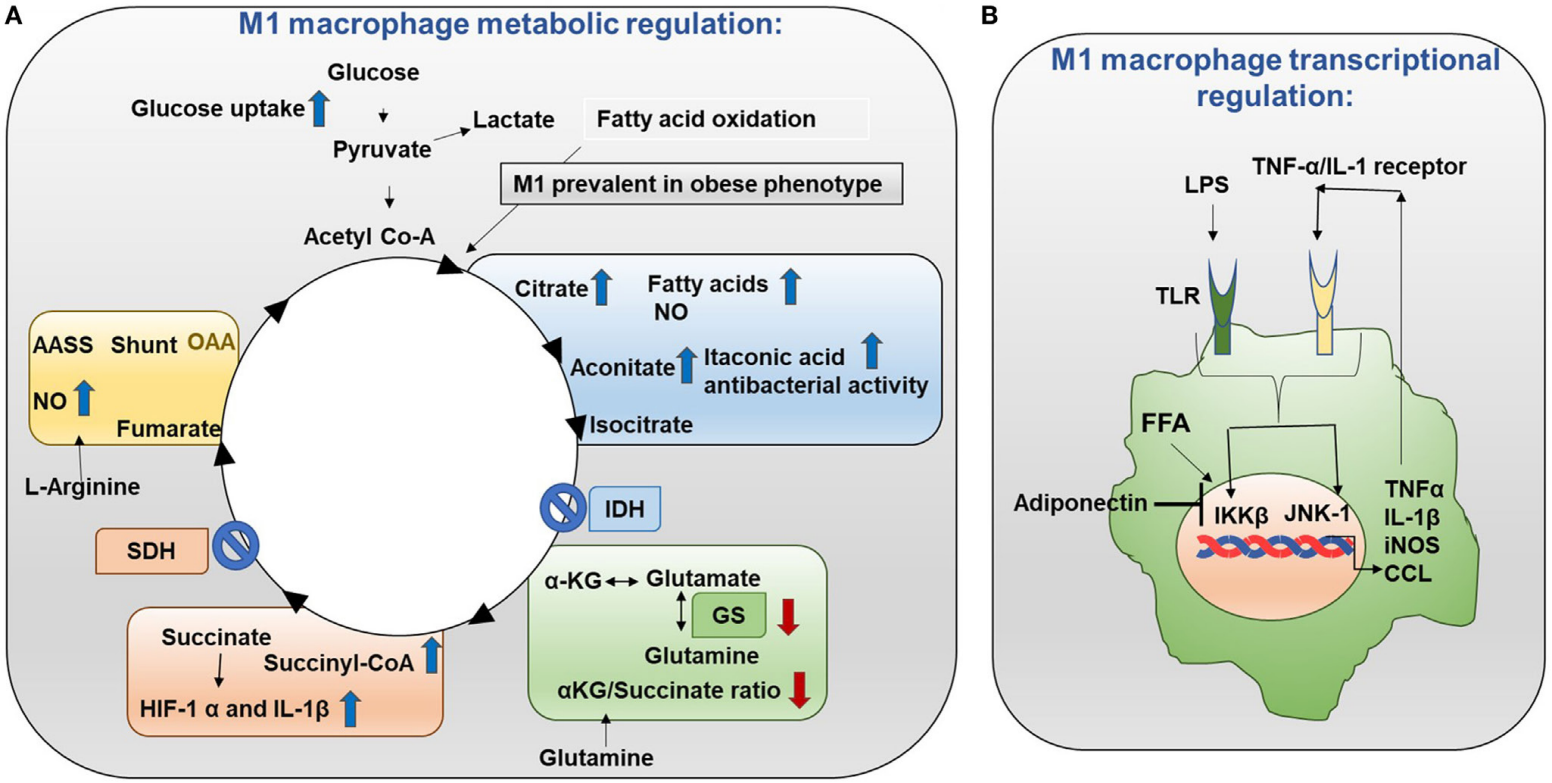
Metabolism in M1 macrophages. **(A)** M1 macrophage metabolic regulation: M1 macrophages are prevalent in obese adipose tissue. Glucose uptake is increased in M1 macrophages. Importantly, the TCA cycle exhibits two breaks. The first break involves the enzyme isocitrate dehydrogenase (IDH) which results in increased levels of citrate and itaconic acid. Citrate feeds fatty acid (FA) synthesis for prostaglandin (PG) and nitric oxide (NO) production while itaconic acid has anti-bacterial properties. The second break happens with the enzyme succinate dehydrogenase (SDH) which causes increased succinate levels. Succinate stabilizes HIF-1α which binds to the interleukin (IL)-1β promoter boosting IL-1β production and inflammation. Increased flow through the aspartate arginosuccinate shunt (AASS) replenishes the TCA cycle which further increases citrate levels and feeds the urea cycle which contributes to NO production. Glutamine is converted into glutamate by glutamate synthase (GS). Glutamate can further be converted into α-ketoglutarate (αKG). Low αKG/succinate ratio strengthens M1 macrophage activation. Glutamine-synthetase inhibition skews M2-polarized macrophages toward the M1-like phenotype characterized by reduced intracellular glutamine and increased succinate with enhanced glucose flux through glycolysis which is partly related to HIF-1α activation. **(B)** M1 macrophage transcriptional regulation: in M1 macrophages, the TCA cycle is broken. As a result, citrate is converted to free fatty acids (FFAs). FFAs activate macrophage IKK and JNK1 signaling molecules to induce M1 polarization. Activation of IKK and JNK1 in macrophages produces IL-1β and TNFα which can initiate inflammatory response. Adiponectin is released from adipose tissue and is involved in the breakdown of fatty acids. Adiponectin signaling inhibits M1 programming.

Metabolic events are tightly controlled in M1 and M2 macrophages. Mechanistically in M1 macrophages, TCA (tricarboxylic acid) cycle exhibits two breaks (Figure 3A) (10, 11). *First break* occurs in the enzymatic step involving isocitrate dehydrogenase (IDH). This results in increased citrate and itaconic acid levels. Citrate is the precursor for fatty acid (FA) synthesis, prostaglandin (PG), and nitric oxide (NO) production. Itaconic acid has anti-bacterial properties which supports the notion that M1 macrophages have inflammatory function. Interestingly, IDH1 and IDH2 are the enzymes that catalyze decarboxylation of isocitrate to α-ketoglutarate outside and inside of the mitochondria, respectively (12). IDH2 plays a vital role in the formation of NADPH which is critical for ROS balance in the mitochondria (13). *Second break* occurs in the enzymatic step involving succinate dehydrogenase. This causes an increase in the expression of succinate. Succinate stabilizes HIF-1α. HIF-1α binds to the IL-1β promoter and promotes IL-1β production. Increased aspartate arginosuccinate shunt will further increase the flow of the TCA cycle. Therefore, this will increase citrate levels and the urea cycle that contribute to NO production. Inhibition of aspartate aminotransferase inhibits NO and IL-6 in M1 macrophages. Thus, the production of NO, IL-1β, and itaconic acid can promote inflammatory functions (14). Also, glutamine metabolism also impacts TCA cycle in M1 macrophages.

#### TCA Cycle in M2 Macrophages

**Figure 4:**
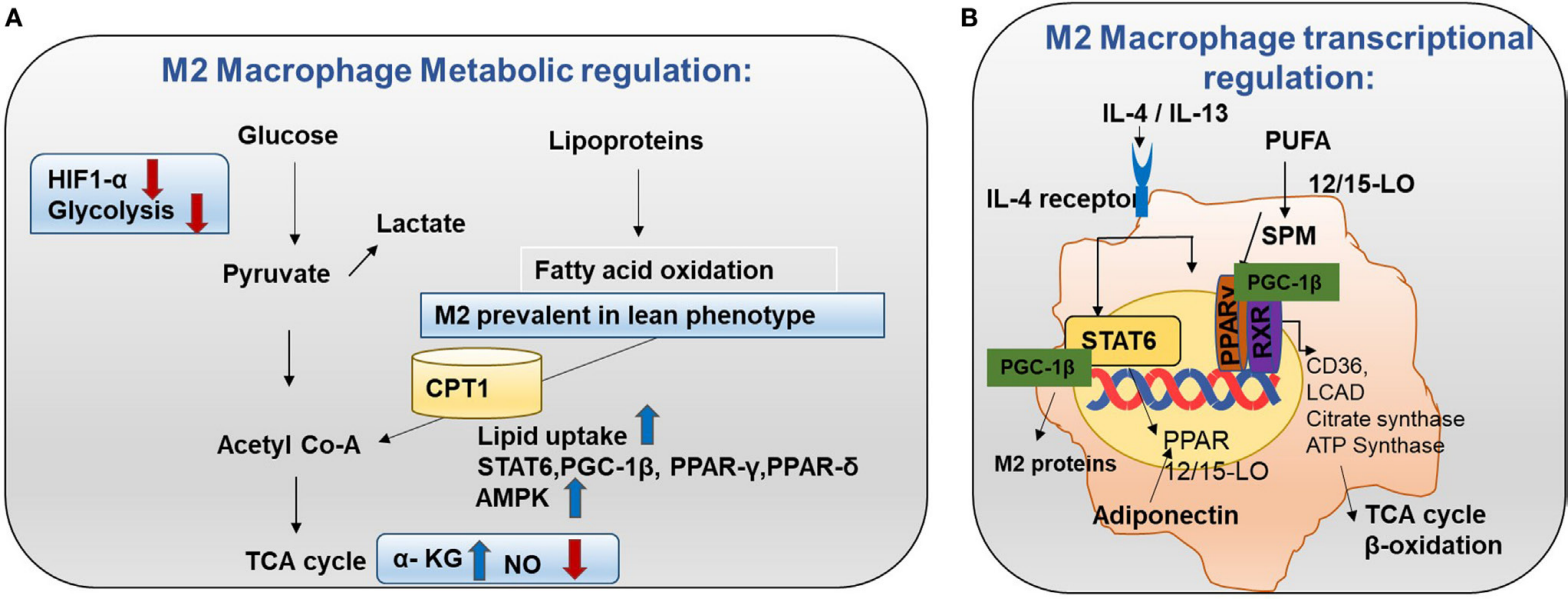
Metabolism in M2 macrophages. **(A)** M2 macrophage metabolic regulation: M2 macrophages adopt a metabolic program fueled by fatty acids and oxidative phosphorylation (OXPHOS). M2 macrophages are seen in lean adipose tissue and lipid uptake is increased in M2 macrophages (8). Both OXPHOS and FAO are important for the anti-inflammatory function of M2 macrophages. In M2 macrophages, glycolysis is impaired due to low HIF-1α activity and low expression of the active 6-phosphofructo-2-kinase/fructose-2,6-biphosphatase 1 (PFKFB1). Also, high α-KG/succinate ratio promotes the M2 phenotype. This could be due to the high flow of the TCA cycle or glutamine metabolism. M2 macrophages will channel FA toward re-esterification and β-oxidation. β-oxidation is channeled through carinitine palmitoyl transferase 1 (CPT1) (9). The high rates of OXPHOS in M2 macrophages are caused by low nitric oxide (NO) production and high adenosine monophosphate-activated protein kinase (AMPK) activity. **(B)** M2 macrophage transcriptional regulation: omega-3 polyunsaturated fatty acids (PUFA) undergo enzymatic transformation by 12/15-LO (lipoxygenase) and other enzymes in the production of specialized pro-resolving lipid mediators (SPMs) and intermediate metabolites that are potent PPAR ligands. M2-inducing cytokines IL-4 and IL-13 promote STAT6 phosphorylation to induce transcription of PPARs (γ and δ) and their coactivator PPARγ-coactivator-1β (PGC-1β). M2 exhibit enhanced PPAR-γ activity. PPAR-γ forms a heterodimer with RXR to induce OXHOS regulating proteins. PGC-1β promotes the STAT6 transcription complex and expression of M2 proteins such as arginase-1 and the pattern-recognition/endocytic receptor CD206. The STAT6 transcription complex further increases transcription of TCA cycle enzymes such as citrate synthase, CD36 (fatty acid transporter), ATP synthase, 12/15 lipoxygenase (converts omega-3 PUFA to SPMs), and LCAD (long chain acyl dehydrogenase, an enzyme for β-oxidation). Adiponectin activates PPARs in M2 macrophages and consequently increases β-oxidation.

M2 macrophages adopt a metabolic program fueled by fatty acids and OXPHOS. Low NO production and high adenosine monophosphate-activated protein kinase (AMPK) can be the reason for the high rate of OXPHOS in M2 macrophages (Figure 4A) (15). Knockdown of pyruvate dehydrogenase kinase 1 diminishes M1 activation whereas it enhances M2 activation of macrophages (16).

#### OXPHOS in Adaptive Immune Cells

Naïve and regulatory T cells exhibit OXPHOS as their primary method of respiration while activated T cells depend on glycolysis. Naïve T cells depend on OXPHOS as their primary metabolic pathway. By contrast, activated T cells exhibit higher glycolysis than OXPHOS. After differentiation, Th1, Th2, and Th17 have higher glycolysis than OXPHOS and high mTORC1 activity. By contrast, memory T cells and regulatory T cells undergo AMPK-dependent FAO and have variable mTORC1 (Figure 2B) (17). Interestingly, glutamine is required for T cell proliferation. Thus, glutamine can provide the intermediate metabolites of the TCA cycle after being converted into α-ketoglutarate in a process called glutaminolysis. These metabolites are important for proliferation. In addition, CD4^+^ T cells can grow in glucose depleted media supplemented with sodium pyruvate (18). This suggests that pyruvate can be utilized, and it is adequate for the survival of T cells in the absence of glucose. In addition, it has been reported that mitochondria undergo rapid changes during T cell activation including increasing mitochondrial mass, number, and mtDNA. This further suggests that mitochondrial metabolism may be crucial for T cell activation and proliferation.

#### Lactate Can Act as a Substrate for Mitochondrial Respiration

Lactate dehydrogenase controls a reversible reaction involving the conversion of lactate to pyruvate (19). Lactate is secreted from tumor cells to the tumor microenvironment (TME). This causes acidification of the TME known as acidosis. Acidosis can suppress the proliferation and cytokine production of cytotoxic T cells (20). Along with cytotoxic T cells, macrophages can be influenced by lactic acid through HIF-1α (21). Furthermore, T cell activation requires activation of nuclear factor of activated T cell (NFAT). High concentrations of lactate can inhibit production of the cytokine interferon gamma (IFN-γ) and transcription of NFAT in both NK cells and T cells. Moreover, genetically targeting LDHA in tumors helps to restore T cell infiltration and function (22). Similarly, umbilical cord derived mesenchymal stromal cells can produce a large amount of lactate. This leads to decreased OXPHOS and mitochondrial mass. Ultimately, this can alter the phenotype and function of dendritic cells (DCs) by inducing M2-type gene signature (23). This indicates that lactate concentration affects activation and survival of the immune cells.

In brief, metabolic changes in immune cells are primarily caused by alterations in metabolic pathways induced by transcriptional changes and TME. In M1 macrophages, the TCA cycle is broken with a decrease in OXPHOS. On the other hand, M2 TCA cycle is fueled by β-oxidation with an increase in OXPHOS. Furthermore, mitochondrial metabolism is important for activation of T cells. Acidification due to lactate production can suppress the T cell proliferation and cytokine production. Therefore, this evidence supports that remodeling OXPHOS in immune cells may be an essential component for developing more efficient therapy for autoimmune disease and cancer.

### Fatty Acid Oxidation (FAO) in Immune Cells

#### FAO in Macrophages

*M1 macrophages* are prevalent in obese adipose tissue (24). Unsaturated fatty acids (nitrosylated fatty acid and omega-3 derived fatty acids) polarize macrophages toward the M1 phenotype (25). In addition, glucose uptake is increased in M1 macrophages (10). Hence due to their broken TCA cycle, M1 macrophages tend to store surplus fatty acid (FA) as triacylglycerol and cholesteryl esters in lipid droplets. By contrast, *M2 macrophages* are seen in lean adipose tissue (24), and their lipid uptake is increased (8). Both OXPHOS and FAO are important for the anti-inflammatory function of M2 macrophages. In M2 macrophages, glycolysis is impaired due to low HIF-1α activity and low expression of the active 6-phosphofructo-2-kinase/fructose-2,6-biphosphatase 1. Also, high α-KG/succinate ratio promotes the M2 phenotype. This could be due to high flow of the TCA cycle or glutamine metabolism. M2 macrophages will channel FA toward re-esterification and β-oxidation (Figure 4A) (11, 26–28). β-oxidation is channeled through carinitine palmitoyl transferase 1 (CPT1), but there are not enough studies supporting how CPT1 is regulated in different macrophages.

#### FAO Controls Transcription of M1 Proteins

In M1 macrophages, the TCA cycle is broken. As a result, citrate is converted to free fatty acids (FFAs). Th1 type cytokines and saturated FFAs activate macrophage IKK and JNK1 signaling molecules to induce M1 polarization. Activation of IKK and JNK1 in macrophages produce IL-1β and TNFα which can initiate inflammatory responses. In addition, IL-1β and TNFα can also activate adipocyte IKK and JNK to block insulin signaling resulting in insulin insensitivity (Figure 3B) (11). Adiponectin is released from adipose tissue and involved in glucose metabolism and break down of fatty acids (29). Adiponectin signaling inhibits M1 programming.

#### FAO Controls Transcription of M2 Proteins

Transcriptional changes in M2 can be the root cause for high FAO. Mechanistically, M2-inducing cytokines IL-4 and IL-13 promote STAT6 phosphorylation to induce transcription of PPARs (γ and δ) and their coactivator PPARγ-coactivator-1β (PGC-1β) (30). PGC-1β promotes the STAT6 transcription complex and expression of M2 proteins such as arginase-1 and the pattern recognition/endocytic receptor CD206 (Figure 4B). Subsequently, STAT6 transcription complex further increases transcription of TCA cycle enzymes such as citrate synthase, CD36 (fatty acid transporter), ATP synthase, 12/15 lipoxygenase (converts omega-3 polyunsaturated fatty acids to SPMs), and LCAD (long chain acyl dehydrogenase, an enzyme for β-oxidation) (11). Also, adiponectin activates PPARs in M2 macrophages and consequently increase β-oxidation. In this way, the M2 program is activated. It supports insulin sensitivity by lowering FFA and promoting lean phenotype. Most importantly, these transcriptional changes lead to β-oxidation in M2 macrophages (Figure 4B).

#### FAO in Memory T Cell Development

Memory T cells are critical for long-term immune response against re-infection by the same pathogen which mainly depends on FAO (31, 32). CD8^+^ T cells play a crucial role in immune responses. Upon antigen stimulation, these cells differentiate into T effector and memory cells for long-term immunity. This differentiation process is closely associated with FAO through AMPK, lipolysis, and IL-7. AMPK is a key regulator of FAO. Metformin activates AMPK which enhances memory T cell development in tumor necrosis factor receptor-associated factor 6 (TRAF6)-deficient CD8^+^ T cells (31). AMPK activation is an important factor for memory T cell development. In addition, lipolysis is the hydrolysis process through which stored lipids are converted into FA. Lysosomal acid lipase catalyzes this process. Memory T cells rely on the expression of lysosomal acid lipase to mobilize FA for FAO (32). IL-7 is required for memory T cell activation as well. Glycerol channel aquaporin 9 (AQP9) is utilized for glycerol transport and triglyceride synthesis, and IL-7 induces the expression of AQP9 in virus-specific memory CD8^+^ T cells (33). AQP9-deficient memory T cells showed less survival because of decreased glycerol import, reduced triglyceride synthesis, and consequently limited FAO. This suggests that IL-7-mediated triglyceride synthesis is important for memory T cell development.

#### FAO in Regulatory T Cell Activation

Stimulated CD4^+^ T lymphocytes can differentiate into effector T cells or inducible regulatory T cell. Michalek et al. found that FAO is required for regulatory T cells differentiation. Glucose metabolism is required for effector T cells and is selectively suppressed by FAO (34). Also, AMPK and mTOR-mediated FAO supports Treg differentiation (Figure 2B). Also, *de novo* synthesized fatty acids are important for FAO and OXPHOS in activated plasmocytoid DCs (35).

In brief, M1 macrophages prefer storing FA while β-oxidation is increased in M2 macrophages. In adipose tissue, M2 macrophages are associated with lean and insulin sensitive phenotype (through regulation of STAT6 and PPARs), whereas M1 macrophages are linked to obese and insulin insensitive phenotype (through regulation of IKK and JNK-1). Current evidence supports that adipose tissue activity is linked to M1 or M2 program activation and obesity. Activated memory T cells (through AMPK and AQP9) and pDCs also support FAO. Concisely, the activation of immune cells is closely related to FAO and the cytokines produced by immune cells which also affect transcriptional changes in the surrounding cells.

### Amino Acid Metabolism in Immune Cells

Amino acid metabolism provides the metabolites needed for immune cells growth. l-Arginine supplementation in activated naïve T cells promotes OXPHOS, limits IFN-γ production, and most importantly helps to express memory T cell markers leading to longer cellular survival (36). Similarly, M1 macrophage polarization also requires amino acid metabolism. For example, arginosuccinate synthase is strongly upregulated in M1 macrophages (14). Inhibition of the aspartate–arginosuccinate shunt enzyme GOT1 (glutamic oxaloacetic transaminase) decreases NO production, and IL-6 production which is characteristic of M1 macrophages.

*SHMT2 (serine hydroxymethyltransferase 2) catalyzes glycine synthesis from serine in mitochondria* SHMT2 is upregulated in activated CD4^+^ T cells (37). SHMT2-deficient activated T cells have decreased purine levels compared with wild-type controls as well as increased DNA damage and impaired survival. Furthermore, SHMT-2 is the primary source for glycine which is important for glutathione synthesis.

#### Tryptophan Metabolism Is Important for Immune Cell Response

**Figure 5:**
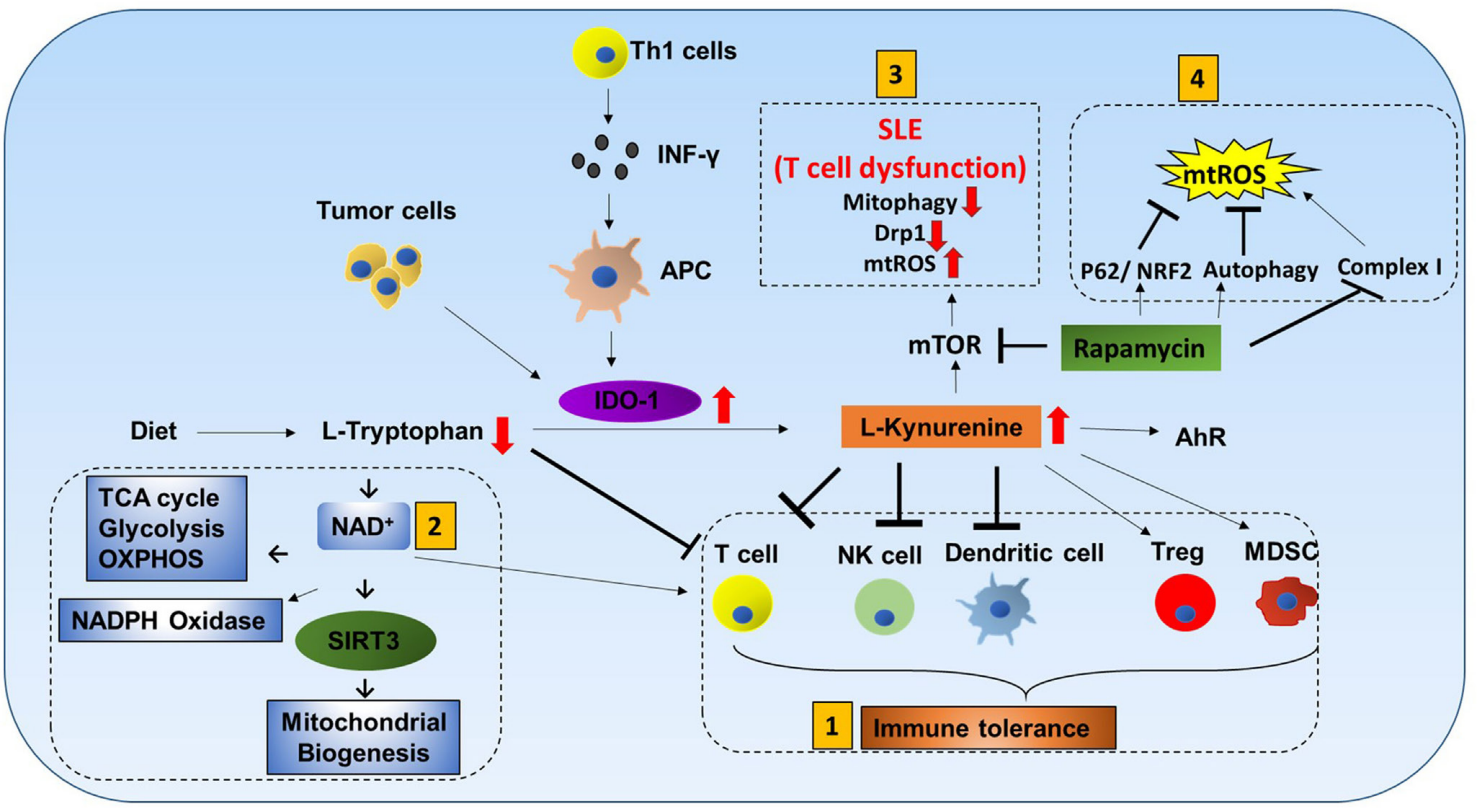
Tryptophan metabolism mediated immune cell response. L-tryptophan (Trp) can only be obtained in the human body from dietary sources. It is catabolized by the enzyme indoleamine-2,3-dioxygenase-1 (IDO-1) to L-kynurenine (Kyn). Interferon-γ produced by Th1 cells stimulates IDO-1 production in antigen-presenting cells (APCs). Tumor cells are also known to have increased production of IDO-1. Increases in IDO-1 cause conversion of Trp to Kyn resulting in lower Trp and higher Kyn in plasma. Kyn is a ligand for aryl hydrocarbon receptor (AhR). Mitochondrial AhR (MitoAhR) is localized in the inner membrane space of mitochondria and is also affected by Kyn. (1) *Immune tolerance by kynurenine*: increased Kyn causes immune tolerance by inhibiting proliferation of both T cells and NK cells by inhibiting maturation of dendritic cells and increasing proliferation of immune suppresser cells (Treg and MDSC). Tryptophan depletion also causes apoptosis of T cells due to nutrient deficiency. (2) *NAD^+^ mediated immune function*: in addition, Trp is one of the sources for NAD^+^ biosynthesis. NAD^+^ is an important metabolite for metabolic processes [glycolysis, TCA cycle, and oxidative phosphorylation (OXPHOS)] and production of NADPH. During oxidative burst in macrophages, NAPPH is oxidized in the presence of NADPH oxidase to produce superoxide which is utilized to kill pathogens. NAD^+^ contributes to CD4^+^ T cell differentiation and SIRT3-mediated mitochondrial biogenesis. (3) *mTOR inhibitor rapamycin can be used to treat systemic lupus erythematosus (SLE) and mitochondrial disease*: T cell dysfunction is associated with SLE. In SLE, an increase in kynurenine and kynurenine mediated mTOR stimulation was observed. In addition, accumulation of reactive oxygen species (ROS) producing mitochondria and decreased Drp1 are associated with SLE. Rapamycin can increase Drp1 by inhibiting mTORC1. (4) *Rapamycin inhibits mitochondrial ROS* (*mtROS*): rapamycin inhibits mtROS through several mechanisms. Complex I in mitochondria is a source of ROS generation. Rapamycin can inhibit mtROS production by inhibiting complex I formation. In addition to this, rapamycin can inhibit mtROS by inducing autophagy and activating p62 and the NRF2 pathway.

l-tryptophan (Trp) cannot be produced by the human body and must be obtained from dietary sources. It is catabolized by indoleamine-2,3-dioxygenase-1 (IDO-1) to l-kynurenine (Kyn). Interferon-γ produced by Th1 cells stimulates IDO-1 production in antigen-presenting cells (APCs), and tumor cells are known to express high levels of IDO-1 (38, 39). Increased IDO-1 causes conversion of Trp to Kyn resulting in a decrease of Trp and an increase of Kyn in plasma. Kyn is a ligand for aryl hydrocarbon receptor (AhR). Increased Kyn causes immune tolerance by inhibiting proliferation of T cells and NK cells, inhibiting maturation of DCs, and increasing proliferation of immune suppresser cells (Treg and MDSC). Also, tryptophan depletion causes apoptosis of T cells due to nutrient deficiency (Figure 5) (40–43).

#### Tryptophan Also Affects Mitochondrial Function

Tryptophan metabolism partially occurs in mitochondria. Trp is one of the sources for NAD^+^ biosynthesis (Figure 5). NAD^+^ is an important metabolite for metabolic processes such as glycolysis, TCA cycle, and OXPHOS (44). During macrophage oxidative burst in the presence of NADPH oxidase, NADPH is oxidized to produce superoxide that is utilized to kill pathogens. Furthermore, NAD^+^ and AhR are also linked to mitochondria. Mitochondrial AhR is localized in the inner membrane space of mitochondria which is also affected by Kyn (45). NAD^+^ has recently gained much interest because CD4^+^ T cell differentiation is controlled by NAD^+^ (46). NAD-dependent deacetylase Sirtuin 3 (Sirt3) is localized in mitochondria. SIRT3 controls mitochondria biogenesis and ATP production. Sirt3 can regulate IDH2 (TCA cycle), LCAD (β-oxidation), and MnSOD (mtROS balance) (Figure 9.1) (47). Furthermore, Sirt3 is dependent on NAD^+^ (Figure 1.2) (48). This suggests that tryptophan metabolism is important for immune response, immune tolerance, and metabolism in mitochondria (Figure 5).

#### mTOR Inhibitor Rapamycin Can Be Used to Treat Systemic Lupus Erythematosus (SLE) and Mitochondrial Disease

T cell dysfunction is associated with SLE. In SLE, an increase in kynurenine and kynurenine-mediated mTOR stimulation was observed (49). When SLE patients were treated with rapamycin (mTORC1 inhibitor), progressive improvement was observed after 12 months in the clinical trial (50). Rapamycin treatment was also effective for mitochondrial diseases such as mitochondrial myopathy (51). In addition, accumulation of ROS producing mitochondria and decreased Drp1 are associated with SLE. Rapamycin can increase Drp1 by inhibiting mTORC1.

#### Rapamycin Inhibits mtROS

Rapamycin inhibits mtROS through several mechanisms. Complex I in mitochondria is a source of ROS generation. Proper assembly of various subunits of complex I is important. Rapamycin can inhibit matrix subunits (NDUFS3 and NDUFV2) as well as prohibitin (PhB) from binding to other membrane subunits (NDUFA9 and NDUFB9). Thus, rapamycin can inhibit mtROS production by inhibiting complex I formation (52, 53). In addition to this, rapamycin can inhibit mtROS by inducing autophagy and activating p62 and the NRF2 pathway (54).

#### Glutamine Metabolism Is Associated With Macrophage Polarization and T Cell Activation

α-Ketoglutarate is an intermediate product of the TCA cycle and is also produced by glutamine metabolism (Figure 3A). αKG is important for alternate M2 macrophage activation by FAO (Figure 4A). Low αKG/succinate ratio strengthens M1 macrophage activation and high αKG/succinate ratio promotes the M2 phenotype (55). In addition, glutamine-synthetase inhibition skews M2-polarized macrophages toward the M1-like phenotype characterized by reduced intracellular glutamine and increased succinate with enhanced glucose flux through glycolysis which is partly related to HIF-1α activation (56). Glutamine is also implicated in T cell functions. SNAT1 and SNAT2 (glutamine transporters) are increased in T cell activation (5). Lack of glutamine inhibits oxygen consumption in effector T cells and reduces ATP concentration. After LPS treatment of macrophages, glutamine metabolism was increased (15). Glutamine deficiency reduces lipid-induced lysosomal dysfunction, inflammasome activation, and cell death in macrophages but boosts autophagy (57).

Taken together, amino acid metabolism especially l-arginine, l-glutamine, l-tryptophan, and SHMT-2 (serine/glycine metabolism) plays a decisive role in the activation of T cells and macrophages.

## Mitochondria Associated Cell Signaling in the Immunity

Mitochondria are a metabolic hub in the cell which functions to meet cellular needs. Obviously, this necessitates that mitochondria receive signals to alter their functions. However, accumulating evidence suggests that mitochondria not only receive signals but also actively provide signals. Mitochondria release proteins, lipids, metabolites, and ROS which can be used as signaling molecules. This crosstalk may coordinate cell-fate decisions and metabolic capacity depending on the cellular environment. Thus, we propose that mitochondria are an integral part of the decision-making process when cells receive immune cues.

### Mitochondrial ROS

Mitochondria are hubs in cellular signaling and produce ROS that drive production of inflammatory cytokines and play a role in removing bacteria. Major sites of ROS production are complex I and III of electron transport chain (ETC) (Figure 9.1).

#### mtROS Contribute to Macrophage Polarization

H_2_O_2_ is released from mitochondria being the source for mtROS. He et al. reported that Cu-Zn SOD-mediated mtROS contribute to macrophage polarization (Figure 9.1). Cu-Zn SOD mediated H_2_O_2_ suppresses M1 phenotype and promotes M2 phenotype. This suggests that mtROS contribute to macrophage polarization (8, 15, 58).

#### mtROS Are Important for the Anti-Bacterial Activity of Immune Cells

Recent studies demonstrate that mtROS significantly contribute to the bactericidal activity and activation of M1 macrophages (59, 60). West et al. found that TRAF6 is recruited to mitochondria resulting in increased mitochondrial and cellular ROS generation. In addition, mitochondrial catalase functions as an antioxidant to control cellular ROS. Mitochondrial catalase transgenic macrophages are less effective at clearing bacteria. This evidence suggests that mtROS are important for the anti-bacterial functions of macrophages.

#### mtROS Contribute to NLRP3 Inflammasome Activation

**Figure 6:**
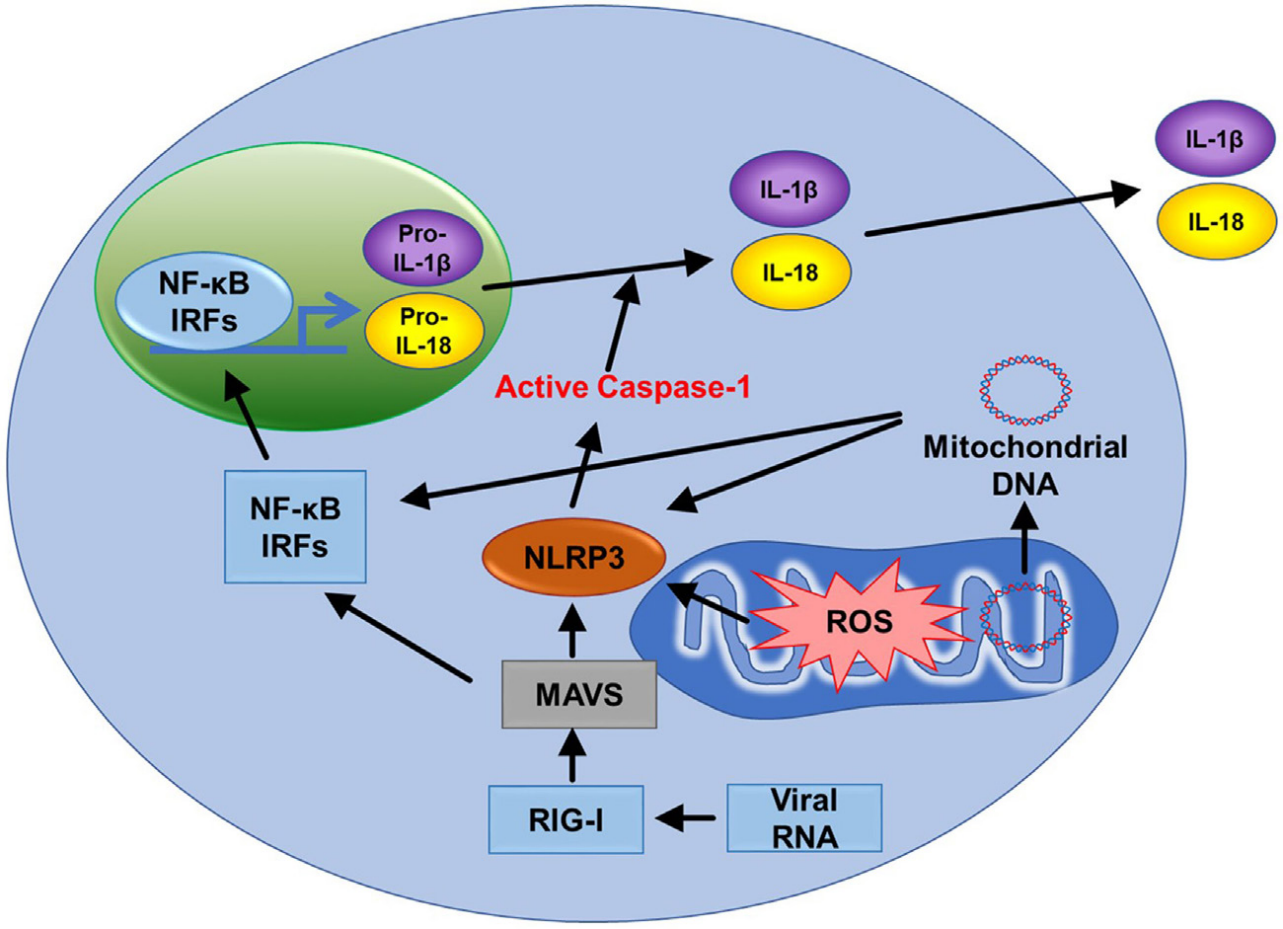
Mitochondria related signal cascades to activate immunity. Mitochondrial antiviral signaling (MAVS) is activated by the viral RNA sensor RIG-I and promotes inflammatory and immune gene expression *via* transcription factor NF-κB and IRFs. MAVS receptors can be found in the mitochondrial outer membrane. Mitochondrial ROS (mtROS) are also able to activate MAVS independently of RNA. The NLRP3 inflammasome also signals from the outer mitochondrial membrane. Like MAVS, NLRP3 also responds to mtROS and can cause mitochondrial damage that promotes the generation of additional ROS. NLRP3 drives the production of IL-1β and IL-18. Mitochondrial DNA can also activate NLRP3. Finally, MAVS promotes oligomerization of NLRP3 at the mitochondria. Therefore, two major innate immune pathways (RIG-I/MAVS and NLRP3) are all dependent on mitochondria.

The NLRP3 inflammasome is a cytosolic complex that plays a key role in innate immunity by participating in the production of pro-inflammatory cytokines interleukin-1β (IL-1β) and IL-18 (Figure 6) (61). The role of the NOD-like receptor (NLR) family of cytoplasmic pattern-recognition receptors in the initiation of inflammatory responses is gathering clear evidence. Structurally, NLRs typically consist of a variable N-terminal effector domain (PYD), a constant central nucleotide binding and oligomerization (NACHT) domain, and C-terminal leucine-rich repeats (Figure 1.6). Upon cellular stress, NLRP3 oligomerizes and exposes its effector domain for interaction with the adaptor protein ASC (apoptosis-associated speck-like protein containing a caspase recruitment domain) and then the complex recruits pro-caspase-1. Pro-caspase-1 clustering leads to its activation and then active caspase-1 cleaves a variety of cytoplasmic targets including IL-1β. In the last decade, several studies found that ROS supplied by mitochondria are essential in activating the inflammasome (2, 62). Nakahira et al. also found that damaged mitochondria in macrophages treated with NLRP3 activator produced high mtROS and then activated the NLRP3 inflammasome (62). In another study, inhibition of mitochondrial complex I- or III-mediated mtROS activated the NLRP3 inflammasome indicating mtROS can be a main source of NLRP3 inflammasome activation (Figure 1.3) (63). It suggests that mitochondria contribute to immune response *via* the NLRP3 inflammasome.

#### mtROS Are Important for T Cell Activation

Stimulation of the T cell receptor (TCR) drives T cells into rapid proliferation and differentiation. T cell activation induces a rapid increase in mtROS production (18). Sena et al. also found that mtROS from complex III are required for CD4^+^ T cell activation and mitochondrial targeted antioxidant mitovitamin E attenuates IL-2 production. An alternative source of mtROS in mitochondria is mitochondrial glycerol-3-phosphate dehydrogenase 2 (GPD2). GPD2 oxidizes glycerol-3-phosphate to dihydroxyacetone phosphate leading to a hyper-reduced state of ubiquinone called ubiquinol in the inner mitochondrial membrane. Kamiński et al. found that GPD2 could directly produce ROS and accumulating ubiquinol could support ROS production at other ETC sites such as complex I (64). GPD2 depletion has been shown to inhibit mtROS production during T cell activation and decrease IL-2 expression. Mitochondrial and GPD2 induced ROS are important for T cell activation.

#### mtROS Also Function as a Signal in B Cell Activation and Antibody Production

Naïve B lymphocytes undergo diversification of their antigen receptor through somatic hyper-mutation, alteration of immunoglobulin function by class-switch recombination (CSR), and differentiation into antibody-secreting plasma cells (PCD) or memory B cells. Bach2 is a transcriptional factor which is required for antigen class switch in B cells. mtROS block heme synthesis in CSR, but it promotes heme synthesis in PCD. As a result, mtROS are promoted during CSR and suppressed during PCD. In support of this, mitochondrial mass and membrane potential are increased in CSR and decreased in PCD (65, 66). Hence, mtROS modulate B cell function by changing heme synthesis.

In short, mtROS are important for immune cell activities in T cells (mediated by GDP2), in macrophages (*via* TRAF6, ECSIT), and in B cells (*via* heme synthesis).

### Mitophagy

Mitophagy is a form of autophagy that specifically eliminates damaged mitochondria for mitochondria quality control. Mitophagy is regulated by PTEN-induced putative kinase 1 (PINK1) and the ubiquitin ligase parkin (67). On the surface of mitochondria, PINK1 recruits parkin from the cytosol and activates parkin’s E3 ligase activity (Figure 1.4). Parkin leads the ubiquitin conjugation of various outer membrane proteins to induce mitochondrial engulfment by an autophagosome followed by subsequent fusion with a lysosome for the clearance of damaged mitochondria.

#### Interruption in Mitophagy Leads to Increased Susceptibility to Pathogens

Roles for mitophagy during pathogen infection have been identified. Degradation of those mitochondria damaged by *Pseudomonas aeruginosa*-produced siderophores requires PINK1 in *C. elegans* (68). On the other hand, parkin mutation showed increased susceptibility to the intracellular pathogenic bacteria *Mycobacterium leprae* and *Salmonella enterica* which causes leprosy and typhoid fever, respectively (69–71). In addition, parkin-deficient mice and flies showed increased susceptibility to *Mycobacterium tuberculosis*, and bacteria proliferation was increased in macrophages (72).

Decreased Mitophagy in T Cell Increases ROS and Apoptosis. T cells utilize mitophagy to maintain their proper homeostasis. Autophagy-related protein 7 (Atg7) is required for the formation of autophagosomes.

Pua et al. found that Atg7-deficient T cells had enhanced mitochondrial content, increased ROS production, and expression of pro-apoptotic proteins like Bak, cytochrome *c*, and AIF (73).

In addition, vacuolar protein sorting 34 (Vps34) is a member of the class III PI3K family of lipid kinases. Vps34-deficient CD4^+^ and CD8^+^ T cells had increased amounts of ROS, mitochondrial mass, and impaired mitophagy (74).

#### Hepatitis B and C Viruses (HBV and HCV) Utilize Mitophagy to Their Benefit

Mitochondria also initiate apoptosis signals. HBV and HCV induce mitophagy to protect themselves from apoptotic signaling. HCV induces parkin and PINK1 leading to mitophagy and mitochondrial dysfunction (75). HBV was found to promote its replication in the cells by promoting PINK1/parkin mitophagy and fission to prevent pro-apoptotic stimuli (76).

Hence, proteins such as PINK1, parkin, ATG7, ATG5, and Vaps34 are important for mitophagy and are implicated in immune cell function. Absence of these proteins may increase the susceptibility to microbial infections and apoptosis of immune cells. Targeting these proteins can benefit HPV and HCV related infections.

### Mitochondrial DNA

NLRP3 inflammasome facilitates activation of caspase-1, secretion of IL-1β and IL-18, and cell death (Figure 6). There are many factors that involve activation of NLRP3 inflammasome such as NF-κB, pathogen-associated molecular patterns, ATP, and potassium ion channels (77). Interestingly, mitochondrial apoptotic signaling can also activate the NLRP3 inflammasome. During mitochondrial dysfunction, mtDNA is released from mitochondria and into the cytosol. mtDNA can bind and activate the NLRP3 inflammasome (Figure 6) (62, 78). In addition, autophagic protein deficiency in macrophages promotes mitochondria dysfunction and consequent release of mtDNA into the cytosol and activation of the NLRP3 inflamasome. Collectively, this suggests that mtDNA mediated activation of NLRP3 is related to autophagy and immune responses. In contrast to this, Allam et al. found that mitochondrial apoptosis was not required for the NLRP3 inflammasome activation, but caspase-8-mediated apoptosis is required for its activation. Nevertheless, this report supports that dysfunctional mitochondria cause mtDNA release and activation of the NLRP3 inflammasome (Figure 1.7) (79). This gives the indication that mtDNA is necessary for NLRP3 inflammasome activation and mitochondrial apoptosis. In addition, high frequency of polymorphisms in the D loop region of mtDNA is observed in lymphocytes of immune-related pancytopenia patients (80). This suggests that mtDNA contribute to red and white blood cell development. How mtDNA is controlled in the case of leukemia or other blood cancers is still not clear.

### Mitochondrial Antiviral Signaling

Mitochondrial antiviral signaling (MAVS) is a signaling protein located on the outer membrane of mitochondria. It is activated by a viral RNA sensor called retinoic acid-inducible gene I (RIG-I) (Figure 6). It activates pathways that regulate NF-κB and interferon regulatory transcription factors (IRFs) to promote gene expression (81). mtROS can drive MAVS oligomerization and production of type I interferon (82) highlighting that MAVS might be a key sensor of mtROS. Furthermore, MAVS associates with NLRP3 and promotes its oligomerization leading to caspase-1 activation (83). Recently, Hee and Cresswell found that MAVS directly interacts with the antiviral protein viperin acting as an immune defense mechanism against RNA viruses in macrophages (84). These studies support that MAVS is activated upon viral infections, induces immunogenic signaling/apoptosis, and mediates the effects of mtROS (Figure 1.5).

## Mitochondrial Dynamics Plays a Key Role in Immune Cell Metabolism

**Figure 7:**
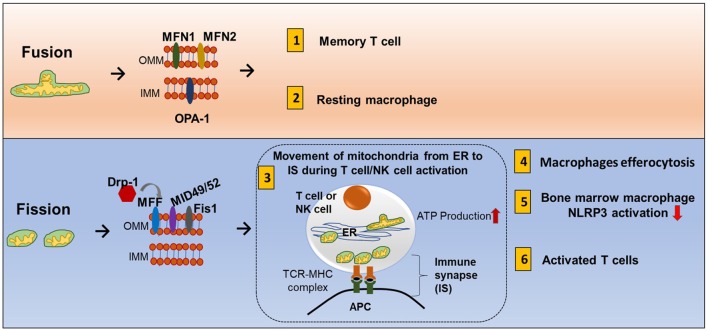
Mitochondrial fission and fusion contribute to immune responses. Mitochondrial fission and fusion controls mitochondrial mass. Nutrient deprivation induces an increase in mitochondrial fusion and suppression of mitophagy. On the other hand, prolonged DNA damage leads to mitochondrial fission. *Fusion (tubular mitochondria)* is controlled by two dynamin-like GTPases for the outer membrane proteins (mitofusin MFN1 and MFN2) and inner membrane proteins (OPA1). *Fission (fragmented mitochondria)* is coordinated by four outer membrane proteins (Drp1, Mff, Mid49, and mid52). Fusion and fission of mitochondria contribute to immune response. (1) Memory T cells have increased fusion generating more oxidative phosphorylation (OXPHOS) and FAO. (2) Clearance of apoptotic cells by phagocytes is known as efferocytosis. Resting macrophages have displayed tubular mitochondria. (3) Fission contributes to mitochondria mobility. During antigen-specific T cell and NK cell activation, mitochondria fission increased, and mitochondria move toward the immune synapse (IS) and facilitate ATP production at the IS. (4) Uptake of apoptotic cells by macrophages during efferocytosis requires fission and increased Drp1. (5) In mouse-derived bone marrow macrophages, knocking down Drp1 initiates NLRP3 inflammasomal activation, and inducing mitochondrial fission attenuated NLRP3 inflammasomal assembly and activation. (6) Activated effector T cells have increased mitochondrial fission and exhibit higher rates of glycolysis.

Mitochondrial fission and fusion controls mitochondrial mass (85). Nutrient deprivation induces an increase in mitochondrial fusion and suppression of mitophagy. On the other hand, prolonged DNA damage leads to mitochondrial fission (86, 87). Mechanistically, fusion is controlled by two dynamin-like GTPases: mitofusin (Mfn1 and Mfn2) for the outer membrane and optic atrophy 1 (OPA1) for the inner membrane. Outer membrane fission is regulated by dynamin-related protein-1 (Drp1), mitochondria fission factor (MFF), Mid49, and mid52 (Figure 7) (88).

The mitochondrial fission and fusion process can have three cellular functions. *First*, it allows mixing of mtDNA content. Each individual mitochondrion has mtDNA that encodes for respiratory complexes, but DNA containing harmful mutations can be pathogenic. A high load of pathogenic DNA can attenuate respiratory function. Fission and fusion can compensate for this problem by mixing mtDNA in different compartments until the pathogenic content becomes too overwhelming. *Second*, smaller particles can move more efficiently so mitochondrial fission can promote mitochondrial mobility inside the cell, and fusion can promote tethering to other cellular structures such as the ER (89). *Third*, mitochondrial fusion can increase cristae formation and can provide more surface area for OXPHOS and FAO while fission eliminates dysfunctional mitochondria and is an adaptation for increased aerobic glycolysis.

During antigen-specific T cell and NK cell activation, mitochondria mobilize toward the immune synapse (IS) (Figure 7). Drp1, a fission factor, is required for translocation of the mitochondria (90, 91). Polarization of cytoskeletal structures, proteins, cell organelles, and transport of ions across membranes at the IS all require energy. This energy is provided by mitochondria. In addition, ATP is also released locally at the IS contributing to T cell function (92). Mitochondrial tethering is also associated with STIM-1 membrane trafficking from the ER to the plasma membrane in mast cells (93). Clearance of apoptotic cells by phagocytes is known as efferocytosis. Resting macrophages have displayed tubular mitochondria. Uptake of apoptotic cells by macrophages requires fission and increased Drp1 (Figure 7) (94). However, there is need of more research to know how fusion and fission regulate macrophage polarization.

### Drp1 Influences ROS Production

Interestingly, Opa1 is deactivated upon ROS increase. Drp1 is activated and causes the fragmentation of mitochondria in neuronal cells (95). This is linked to iron overload, AMPK activation, MFF (96), and ubiquitination of A-kinase anchor protein 121 (97). Conversely, ROS induced mitophagy is suppressed by depleting Drp1 (98). In addition, accumulation of mitochondria and decreased Drp1 is associated with T cell dysfunction in SLE (SLE → T cell dysfunction → mitochondria accumulation → Drp1 depletion) (99). Rapamycin can increase Drp1 by inhibiting mTORC1 (100). This means that Drp1 can be targeted in ROS producing mitochondria and other immune diseases.

### Fusion and Fission in Diverse Immune Cells

Activated effector T cells have an increase in mitochondrial fission and mitochondrial mass but less cristae formation to support aerobic glycolysis. Memory T cells have an increase in fusion which consequently causes OXPHOS and FAO to be increased (Figure 1.8) (101). The receptor-interacting protein kinase 3 (RIPK3) plays a crucial role in natural killer T cell (NKT cell)-mediated immune response *via* activation of the mitochondrial phosphatase phosphoglycerate mutase 5 (PGAM5). RIPK3-mediated PGAM5 promotes nuclear translocation of NFAT and dephosphorylating Drp1 in NKT cells (102). In addition, there is some evidence that mitochondrial dynamics is also related to inflammasomal activation. Obese rats have increased expression of Drp1 and NLRP3 and decreased fusion-relative protein optic atropy-1 (OPA1) (103). In mouse bone marrow-derived macrophages, knocking down Drp1 initiates NLRP3 inflammasomal activation, and inducing mitochondrial fission attenuated NLRP3 inflammasomal assembly and activation (104).

In addition to mtDNA mixing, mobility, and metabolic regulation, mitochondrial dynamics also control the inflammasomal activation. However, how the genes for fusion and fission are controlled is still unknown.

## Mitochondria are a Node for ROS, and Antioxidants are Important for the Neutralization of ROS

### Antioxidants Play an Important Role in Neutralizing mtROS

In mitochondria, superoxide $\left({\text{O}}_{2}^{-}\right)$ is released from complex I (into the matrix) and III (into both the matrix and intermembrane space). This is converted to H_2_O_2_ in the presence of SOD. Catalase converts H_2_O_2_ to H_2_O. H_2_O_2_ can also be converted to HOCl in the presence of myeloperoxidase. HOCl is produced by neutrophils to damage macromolecules of pathogens and ultimately kill the pathogens. H_2_O_2_ can also go through the fenton reaction to produce highly reactive OH^−^. This can react with different components of the cell and cause damage. Glutathione converts OH^−^ to H_2_O to reduce the cellular damage. Superoxide, OH^−^, and H_2_O_2_ generated in mitochondria are the main sources of ROS. Hence, antioxidants such as catalase, Mn-SOD (manganese dismutase in mitochondria matrix), Cu/Zn-SOD (in the intermembrane space and cytosol), and glutathione play a crucial role by neutralizing the ROS (Figure 9.1) (105).

#### ROS Are Important to Maintain Cellular Homeostasis

Low or moderate concentrations of ROS help cells maintain healthy conditions. For example, oxidative burst in macrophages generated using NADPH oxidase releases H_2_O_2_ during phagocytosis to kill pathogens. H_2_O_2_ is converted into HOCl to kill pathogens by damaging macromolecules. H_2_O_2_ influences mtDNA and nuclear DNA transcription of antioxidant genes (NRF2, AP-1). However, it can lead to pathological conditions in the case of excess of ROS. Excess levels of ROS inhibit glucose transporter proteins and cause alterations in cellular signaling and oxidative damage to macromolecules. Antioxidants neutralize the ROS and maintain healthy conditions (Figure 9.2).

#### Glutathione Metabolism and Function

Glutathione can be considered “mother of the antioxidant defense system.” Glutathione is synthesized from amino acids in a two-step reaction. In the presence of γ-glutamylcysteine synthetase, glutamic acid and cysteine are converted into γ-glutamylcysteine. Glycine is then added in the presence of glutathione synthetase to make glutathione. γ-glutamylcysteine synthetase regulates glutathione synthesis. Oxidative stress influences the function of γ-glutamylcysteine synthetase. Glutathione is oxidized and reduced to glutathione disulfide (GSSG) in a reversible reaction. NADPH from the pentose phosphate pathway reduces GSSG to form glutathione pathway (GSH). GSH is important for conversion of OH^−^ to H_2_O. Mitochondrial GSH also contributes to detoxification of harmful lipids (lipids-OOH) and deglutathionylation of mitochondrial proteins (106).

#### Other Antioxidant Proteins Present in Mitochondria

In addition to glutathione, other antioxidants (Co-Q, SIRT3, FOXOs) also influence ROS. Coenzyme Q (Co-Q) is an essential antioxidant that carries electrons from complex I–II and III. Co-Q is synthesized in mitochondria requiring proteins coded in 12 genes. Mutations in Co-Q genes cause CoQ10 deficiency which leads to an increase in ROS and ATP depletion (107). Sirtuin 3 (SIRT 3) coordinates FAO and superoxide detoxification to control cellular ROS levels by deacetylation of acytyl co-A dehydrogenase, Mn-SOD, and FOXO3 (108).

### Glutathione Depletion Leads to Impairment of Immune Cell Function

#### Glutathione and APCs

Antigen-presenting cells display MHC on their surface which interacts with TCRs on T cells to drive immune function and cytokine production. Glutathione levels within APCs and immune cells regulate their function. GSH depletion in APCs correlates with reduced secretion of Th1 cytokines. Increased intracellular GSH content stimulates IL-12 or IL-27 which in turn differentiates CD4^+^ T cells to Th1 cells (109). In addition, activated macrophages and DCs secrete antioxidant precursors such as cysteine. Cysteine is taken up by T cells. This allows T cells to be protected from harm during antigen presentation.

#### Glutathione in Other Immune Cells

In support of this, glutathione depletion was observed in other immune cells. Decreased GSH/GSSG ratio in CD8^+^ memory T cell leads to an increase in ROS and impairment of CD8^+^ memory T cells (50). IL-17-producing γδ T cells (γδ17 T cells) have recently been found to promote tumor growth and metastasis. Low levels of glutathione are also observed in γδ17 T cells (110). These factors suggest that decreased GSH can disrupt immune cell function.

#### Glutathione During HIV Infection

It is well known that HIV suppresses the immune system. HIV infection is associated with elevated levels of ROS and decreased GSH. However, increasing GSH levels in NK cells reduces intracellular survival of pathogens in macrophages (111). HIV infection destroys CD4^+^ T cells. Supplementation of L-GSH to HIV-positive patients with low CD4^+^ T cell counts resulted in an increase in IL-12, IL-2, and IFN-γ and a decrease in IL-6, IL-10, and free radicals as well as stabilization of the levels of TGF-β, IL-1, and IL-17 (112). This implies that glutathione can impact cytokine production.

#### Glutathione and Post-Translational Modification of Proteins in Immune Cells

Glutathione can also reverse ROS-mediated post-translational modifications. Increased H_2_O_2_ production results in increased protein S-glutathionylation in both monocytes and differentiated macrophages. Protein S-glutathionylation can be prevented either by the activity of antioxidant enzymes at the level of ROS scavenging or reversed by glutaredoxin-mediated deglutathionylation (113, 114).

In short, glutathione plays a vital role in APCs and T cell differentiation, the function of NK cells and macrophages, cytokine production in immune cells, and post-translational modifications of proteins in immune cells.

### Antioxidants Regulate ROS-Mediated Cell Signaling Pathways in Immune Cells

**Figure 8:**
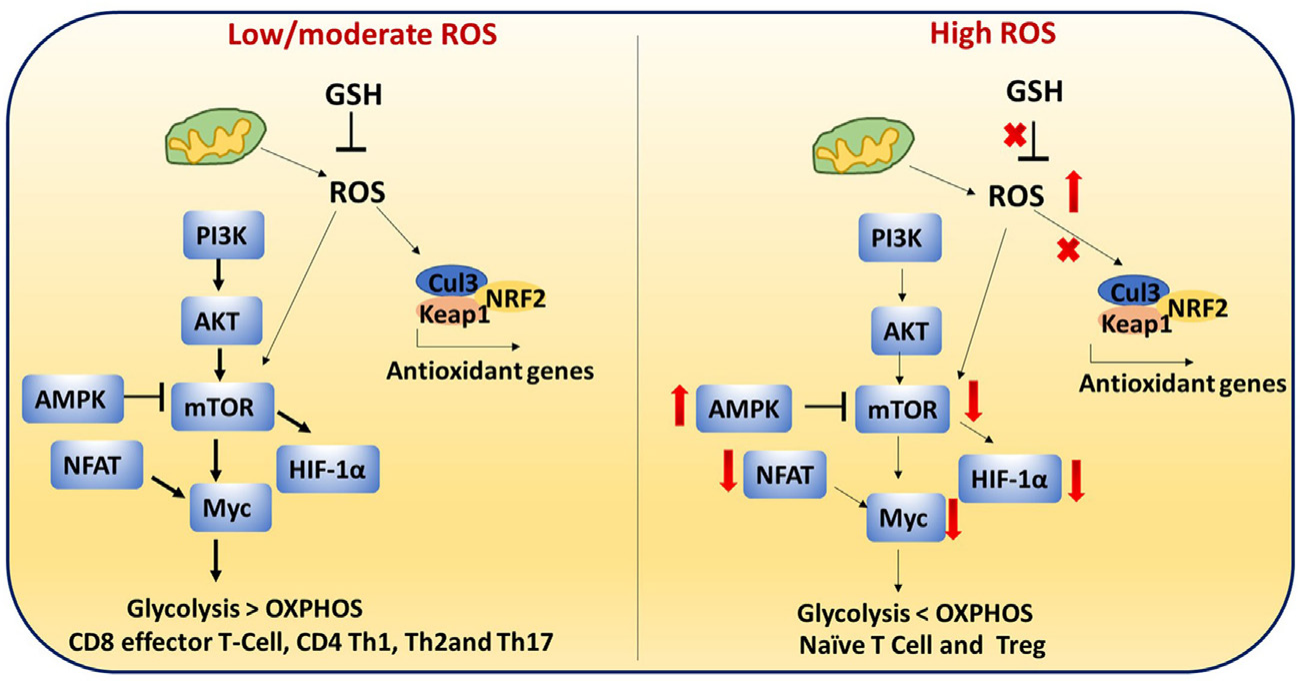
Reactive oxygen species (ROS)-mediated cell signaling in T cells: low/moderate levels of ROS promote mTOR, Myc, and nuclear factor of activated T cell (NFAT) activation which is associated with increased glycolysis and inflammatory response in T cells. ROS levels are regulated by the glutathione pathway (GSH) and NRF2–Keap1–Cul3 trimeric complex. Abrogation of GSH and the trimeric complex, which may cause high levels of ROS, impairs the inflammatory response of T cells. In addition, high ROS and high adenosine monophosphate-activated protein kinase (AMPK) levels inhibit mTOR. It is reported that naïve T cells, CD4^+^ regulatory T cells, and chronically stimulated T cells depend on OXPHOS as their primary metabolism method while CD8^+^ effector and CD4^+^ Th1 cells mainly depend on glycolysis. This metabolic switching is controlled by ROS.

Antioxidants neutralize the cellular ROS levels while also influencing transcription of antioxidant genes and post-translational modifications of proteins involved in antioxidant pathways. Transcription factors such as NRF2 and Keap1 control expression of antioxidant genes. Therefore, ROS levels are regulated by the GSH and NRF2–Keap1–Cul3 trimeric complex. T cell-mediated autoimmune disease is mediated by dysregulation of these pathways and elevated levels of ROS (Figure 8) (115).

**Figure 9:**
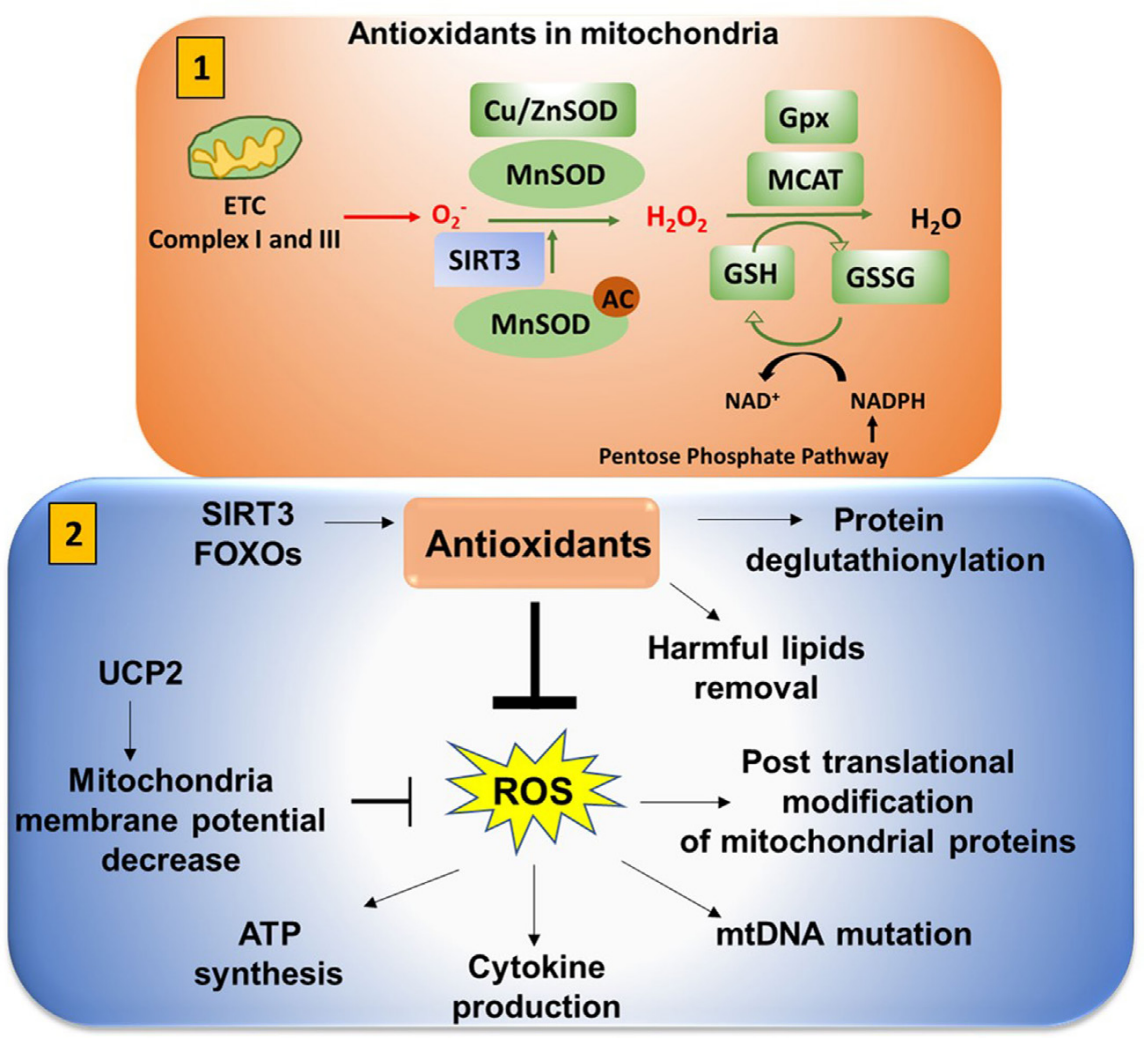
Antioxidants neutralize mitochondrial ROS (mtROS). (1) *Antioxidants in mitochondria*: superoxide $\left({\text{O}}_{2}^{-}\right)$ is released from complex I (into the matrix) and III (into both the matrix and intermembrane space). This is converted to H_2_O_2_ in the presence of superoxide dismutase (SOD). Mn-SOD (manganese dismutase) is localized in mitochondria matrix and Cu/Zn-SOD (in the intermembrane space and cytosol). SIRT3 is involved in deacetylation of MnSOD. Catalase converts H_2_O_2_ to H_2_O. Catalase is also localized in mitochondria (MCAT). Glutathione converts OH^−^ to H_2_O to reduce the cellular damage. Glutathione (GSH) is oxidized and reduced to glutathione disulfide (GSSG) in a reversible reaction. NADPH from the pentose phosphate pathway reduces GSSG to form GSH in presence of glutathione peroxidase (Gpx). (2) *Antioxidants play an important role in neutralizing mtROS*. Uncoupling protein 2 (UCP2) (mitochondrial uncoupler protein), which helps to shuttle H^+^ ions from the intermembrane space to the mitochondria matrix, decreases the membrane potential to inhibit excessive reactive oxygen species (ROS) production. Excessive ROS production can impact ATP synthesis, cytokine production, mitochondrial DNA (mtDNA) mutation, and post-translational modification of cellular proteins by S-nitosylation or glutathionylation.

T cell metabolism is highly dynamic. Proper T cell activation and differentiation is critical (116). It is reported that naïve T cells, CD4 Treg, and chronically stimulated T cells depends on OXPHOS as the primary metabolism method while CD8 effector and CD4 Th1 cells mainly depend on glycolysis (117). This metabolic switching may be controlled by ROS.

During activation, CD4^+^ T cells must transition metabolically from OXPHOS to aerobic glycolysis to support proliferation and effector function. Low/moderate levels of ROS promote mTOR (Figure 8) (118), Myc, and NFAT activation. mTOR activation leads to increased glycolysis in T cells. Abrogation of GSH, which may cause high levels of ROS, impairs the inflammatory response of T cells. High AMPK levels inhibit mTOR and increase ROS (17, 119, 120). High ROS and diminished mTOR activation lead to decreased Myc and reduced transition to aerobic glycolysis in diabetogenic splenocytes. These results suggest that ROS are required for the metabolic transition (121). High levels of ROS and low GSH can lead to improper cellular signaling and post-translational modifications of proteins which can impact metabolic outcome.

### Antioxidants Influence Mitochondrial Membrane Potential (MMP)

Mitochondrial membrane potential (ΔΨm) is generated as protons are pumped outward from the matrix, a process that depends on substrate utilization and electron transport. This is a critical check point for cell death and ATP synthesis. Loss of membrane potential may result from any processes wherein protons move back toward the matrix. UCP2 acts like an antioxidant to reduce ROS by moving protons back to the matrix (122). Sirt3 and FOXO activation decrease mtROS. Similarly, mitophagy through activation of BNIP3 or NIX decreases mtROS by decreasing the number of damaged mitochondria that produce more ROS. ΔΨm plays a decisive role by driving ATP synthesis. ΔΨm is regulated by glutathione which depends on thioredoxin and NADPH generated in the pentose phosphate pathway (123). There is still a need for more research on potassium and calcium channels that also have an impact on MMP (124).

#### Antioxidants Impact on MMP, mtDNA, and ATP Synthesis

T cell activation and proliferation depend on the production of reactive oxygen intermediates (ROIs). Mitochondrial hyperpolarization (MHP) is associated with increased ROI, decreased GSH, and ATP depletion. This MHP is crucial for T cell activation (125–127). In cancer cells, mitochondrial H^+^-ATP synthase is increased and ATPase inhibitory factor 1 is decreased when compared with normal tissues. This in turn promotes ROS production and ATP depletion and further decreases the antioxidant defense system (128).

#### Excess ROS Can Lead to mtDNA Mutation

Reactive oxygen species can also cause mutations in mtDNA and further change MMP and ATP synthesis. mtDNA contains 37 genes. 12 proteins are encoded by mtDNA as structural proteins of mitochondrial enzyme complexes (I-IV). Nuclear genes encode about 1,500 mitochondrial proteins. Changes in cellular ROS levels can cause mutations in mtDNA which further disturbs the normal functions of mitochondrial proteins (105). Mutation load in the ATP6 gene leads to a defect in mitochondrial ATP synthase which can further increase MMP and ATP synthesis depletion (129).

## Does Mitochondrial Immune Regulation have Any Therapeutic Importance?

Based on previous evidence, mitochondria are important for autoimmune disease, mitochondrial disorders, and cancer. Selectively targeting OXPHOS can be effective for advanced melanoma (130). Also, altering mitochondrial machinery in sepsis can increase patient survival by 30% (131). From this, a new field has emerged called “mitochondrial medicine.” Drugs can be developed selectively by targeting them to mitochondria causing a switch in immune programing (132, 133). Here, we have highlighted how antioxidants can be delivered into mitochondria and how it can be useful during ischemia reperfusion.

### Role of Antioxidants in Mitochondria

Antioxidants can be targeted selectively to mitochondria to reduce oxidative stress (134, 135). Natural antioxidants (vitamin E, curcumin, ginko biloba, melatonin) in addition to targeted TPP-based antioxidants (MitoQ, Mito-VitE, Mito-α-lipoic acid, Mito-PBN), small peptide-based molecules (SS31, SS02, SS19, SS20), choline esters of glutathione, and *N*-acetyl-l-cysteine neutralize mtROS which further maintains normal MMP (136, 137). These molecules are preferentially taken up by mitochondria due to differential charge (negative charge in mitochondria and positive charge on the molecules). Antioxidant SS31 has been shown to inhibit fission proteins (Drp1 and Fis1). Antioxidants can be used as targeted therapy for mitochondrial disorders (138). Also, TPP conjugated antioxidants have shown to potently inhibit cancer cell proliferation (139).

### Mitochondrial Components Are Modified by Antioxidants During Ischemia/Reperfusion (IR)

Ischemia/reperfusion injury is the tissue damage caused by the return of blood supply after a period of ischemia leading to a state of hypoxia. ROS are known as the primary cause of ischemic tissue injury. In mitochondria, several protein complexes are modified by S-nitrosylation and S-glutathinylation (140). Complex I is modified by both processes. Complex I has two transitional states: active (A) and deactive (D). Complex I is S-nitrosylated during D state which can be reversed by thiol reductants. Reperfusion of ischemic tissue rapidly activates complex I and increases the generation of ROS which leads to cell death. The presence of MitoSNO (S-nitrosothiol) or S-nitrosylated agents during reperfusion selectively target the ND3 subunit of complex I at Cys39 to keep the complex in low activity and decrease ROS production. In this way, S-nitrosylation of ND3 at Cys39 in complex I protects against IR injury (141–143).

Other mitochondrial components have been shown to treat diseases. However, whether these can also be implicated in immune-related functions is still not known. For example, coenzyme Q10 in mitochondria can be utilized as a potential treatment for heart failure (144). Genipin is a UCP2 inhibitor that is useful for anticancer therapy (145). Circulating mtDNA can be used as potential biomarkers for chemotherapy induced cardiac damage (146). Whether circulating mtDNA and genipin can be useful for leukemia or other immune diseases is still unknown.

## Concluding Remarks

The importance of mitochondria in immunity has become clearer. Besides controlling cell fate, mitochondria provide signaling platforms generated by MAVS, ROS, and mtDNA. Mitochondria balance redox status to fine-tune NLRP3 inflammasome activation. Maintenance of mitochondrial fidelity by mitophagy is important for cell fate and immunity. The protective role of mitophagy has the potential to treat inflammatory diseases with excess ROS and mitochondrial dysfunction. This idea is supported by the observation that mitochondrial antioxidants help ameliorate symptoms (147, 148). In addition, targeting this pathway can be a therapeutic strategy because defective mitophagy has been implicated in Parkinson’s disease.

In addition, mutations in mitochondrial proteins have gained clinical importance. For instance, apoptosis inducing factor mitochondria associated-1 (AIFM-1) is related to mitochondria function and immune system regulation. Mutations in AIFM-1 are related to fatal encephalomyopathy in infants (149). Recurrent mutations in IDH2 are associated with angioimmunoblastic T cell lymphoma (150). Polymorphisms in MnSOD can lead to abortion during the first trimester of pregnancy (151). This evidence supports the conclusion that immunodeficiency is related to disrupted mitochondrial components. However, there is still need of more research to know what leads to these types of mutations/polymorphisms and how they affect immune cell functions.

For immunologists, mitochondria can be the powerhouse of immunity along with their roles as the powerhouse of the cell. After many decades of hard work, remarkable developments in immunology research have improved our understanding of the immune system, and immunologists are now better equipped with modern knowledge and techniques to cross over into other disciplines. Future work will continue to reveal the functions of mitochondria in immunity. For example, “What are the roles of mitochondrial fission and fusion as well as cristae remodeling in immunity?” or “Do mitochondria affect other innate immune cells such as innate lymphoid cells and granulocytes?” We hope this review will inspire research into many questions that remain to be explored. Coupling the unique benefits of studying mitochondria and immunity is beneficial for the enormous clinical relevance in human health and diseases.

## Author Contributions

This manuscript was conceived by MT, designed and written by SL, AA, CY, and MT, and revised by JP, J-HK, and ZY. MT supervised development of this paper as the principal investigator.

## Conflict of Interest Statement

The authors declare that the research was conducted in the absence of any commercial or financial relationships that could be construed as a potential conflict of interest.
